# Detection of *Plasmodium* in *Anopheles* spp (Diptera: Nematocera: *Culicidae*) using molecular biology techniques: a systematic review

**DOI:** 10.1590/S1678-9946202567071

**Published:** 2025-10-13

**Authors:** Erique da Costa Fonseca, Everson dos Santos David, Karen Carmo dos Santos, Franciane Pereira de Oliveira, Ledayane Mayana Costa Barbosa, Shirley Vasconcelos Komninakis, Raimundo Nonato Picanço Souto

**Affiliations:** 1 Universidade Federal do Amapá, Programa de Pós-Graduação em Biodiversidade e Biotecnologia da Amazônia Legal, Macapá, Amapá, Brazil; 2 Universidade Federal do Amapá, Laboratório de Arthropoda, Macapá, Amapá, Brazil; 3 Universidade Federal do Amapá, Programa de Pós-Graduação em Ciências Farmacêuticas, Macapá, Amapá, Brasil; 4 Universidade Federal de São Paulo, Laboratório de Retrovirologia, São Paulo, São Paulo, Brazil; 5 Universidade Federal do Amapá, Programa de Pós-Graduação em Biociências, Macapá, Amapá, Brazil

**Keywords:** Molecular biology, Plasmodium, Malaria, Anopheles

## Abstract

This study aims to find evidence of the effectiveness of molecular biology techniques in detecting *Plasmodium* in *Anopheles spp* mosquitoes. This systematic review was based on the PRISMA 2020 protocol. It was carried out in five electronic databases (LILACS, PubMed, SciELO, Scopus, and Web of Science, with published studies in health and interdisciplinary areas) in addition to complementary research on Google Scholar. Studies that used molecular biology techniques to detect and evaluate *Plasmodium* in anophele*s* (the results of which determined the type of *Plasmodium* in the samples) were included in this review. In total, 484 recent studies were retrieved from the electronic databases. According to the inclusion/exclusion criteria, only 12 studies met the objectives of this systematic review. Molecular biology was used in mosquitoes to determine parasitic species in all studies. Despite the difficulties and challenges in using molecular biology in mosquitoes, the obtained scientific advances show the accuracy and reliability of the results, contributing to an effective epidemiological response and monitoring of the spread of *Plasmodium* in endemic areas.

## INTRODUCTION

Malaria is a serious and potentially fatal infectious disease caused by *Plasmodium* parasites that is transmitted to humans by infected female *Anopheles* mosquitoes. According to global estimates, 2023 witnessed about 263 million cases of the disease, resulting in around 597 thousand deaths in 83 countries^1^.

The areas with the highest prevalence of malaria mainly lie in Sub-Saharan Africa, responsible for 94% of all cases (246 million) and 95% of related deaths (569 thousand)^1^ in 2023. Other endemic regions include parts of Asia, Latin America, and to a lesser extent the Middle East and some areas of Europe. The Americas witnessed 505,600 cases and about 116 deaths in the same period^1^.

In total, five *Plasmodium* species infect humans, of which *P. falciparum* and *P. vivax* show the greatest prevalence and concern. *P. falciparum* stands out as the most lethal and predominant species in Africa, whereas *P. vivax* occurs more often outside sub-Saharan Africa, especially in the Americas and Southeast Asia^1^.

The genus *Anopheles* includes around 490 species of mosquitoes, of which from 60 to 70 can transmit malaria. Of these, about 30 constitute greatly epidemiologically relevant vectors^2^. In Brazil, the species *Anopheles darlingi* stands out as the main vector of the disease in the Amazon^3^.

The accurate identification of *Plasmodium* parasites in *Anopheles* mosquitoes is essential to understand the dynamics of malaria transmission. In this context, molecular biology techniques, such as polymerase chain reaction (PCR), constitute effective tools to detect *Plasmodium* infections in vectors. A recent study, for example, applied PCR to identify 800 *Anopheles* specimens, grouping them into 160 pools, evincing the high effectiveness of this technique in finding infections in vector mosquitoes^4^.

Molecular techniques such as PCR provide high sensitivity and specificity in the detection of *Plasmodium* infections in vectors, finding infections in their early stages or with low parasitemia. Furthermore, these methodologies facilitate the distinction of *Plasmodium* species, offering essential information for more effective and targeted control strategies^5^.

Prospects in the fight against malaria focus on integrated control strategies, such as effective vaccines, improved diagnostic and treatment methods, and strengthened vector control measures. Advances in genomic research have significantly contributed to the understanding of the biology of the parasite and its vector and interaction with humans, opening new possibilities for disease control^6^.

Thus, this systematic review aims to seek evidence of the effectiveness of molecular biology applied to *Anopheles* mosquitoes to study *Plasmodium* diversity in endemic areas and the spread of malaria throughout the world.

## MATERIALS AND METHODS

This systematic review was based on the protocol Preferred Reporting Items for Systematic Reviews and Meta-Analyses 2020 (PRISMA)^7^, which was adapted according to recent systematic review studies^8,9^. It was catalogued in the International Prospective Register of Systematic Reviews database of systematic review protocols under no. CRD42024538083.

The formulation of the guiding question of this research was developed using the acronym PICo as an auxiliary tool^10^. PICo is composed of three components, namely population or problem, phenomenon of interest, and context. In this review, three PICo components—namely, human malaria vectors (problem), the effectiveness of molecular biology techniques (interest), and *Plasmodium* infectivity in *Anopheles* mosquitoes (context)—were used to elaborate the following research question: What is the evidence of the effectiveness of molecular biology techniques in detecting *Plasmodium* in *Anopheles* mosquitoes?

This research was carried out from April to July 2024 in five electronic databases: LILACS, PubMed, SciELO, Scopus, and Web of Science. Studies that were published in health and interdisciplinary areas in English, Spanish, and Portuguese without restrictions on publication date were included. A selection of Health Sciences Descriptors and Medical Subject Headings in English was used as search terms to retrieve articles by their titles and abstracts.

**Figure 1:**
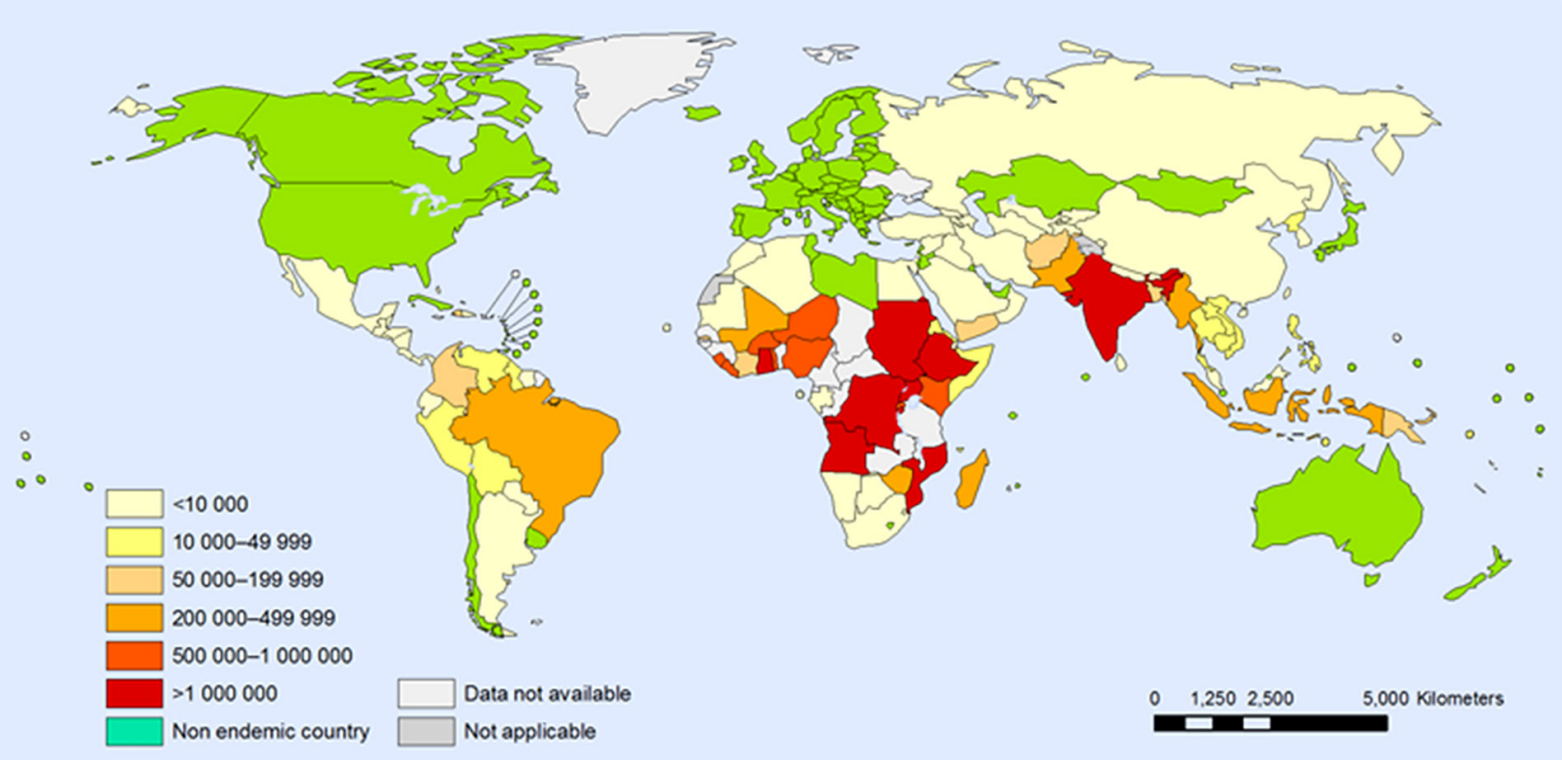
Malaria risk in about 100 tropical and subtropical countries.

The search strategy was carried out using the descriptors in Figure 1. A manual search was also carried out in the references of the selected articles, as was a search on Google Scholar to find relevant non-indexed studies.

### Eligibility criteria

Studies that aimed to detect *Plasmodium* in *Anopheles* mosquitoes (the main vectors of human malaria) by molecular biology (to determine infectivity in anopheles, the results of which showed the dissemination of parasitic species in infected mosquitoes and the following dissemination of malaria) were considered for inclusion in this research.

Primary prospective, retrospective, or cross-sectional studies with experimental designs that were carried out in any region of the world were included in this review. Studies related to study objects such as ticks, blood samples, or other insects (even if analyzed by molecular biology) were excluded.

### Data extraction and evidence synthesis

The retrieved search results in the databases were transferred to the Mendeley reference manager, version 1.18, to remove duplicates. Then, the Rayyan QCRI platform was used to select the articles. The first stage consisted of title and abstract independent analyses by two researchers (ECF and ESD). Disagreements were independently solved by a third researcher (RNPS). Then, two authors (ECF and ESD) independently read the articles in their entirety. Moreover, the reference list of the included studies was searched for potentially eligible studies that were missed in the database search.

Data were independently extracted by two reviewers (ECF and ESD) using an extraction form in Microsoft Excel 2016 (Microsoft, Redmond, WA, USA), the discrepancies of which were solved by consensus with a third researcher (RNPS). The data extraction form for eligible studies included the following topics: *Anopheles* genus, species, year of study, study objective, study area, habitat characteristics, country, mosquito distribution, number of pools, *Plasmodium* and/or malaria in the samples, and the molecular biology technique used in the research.

### Quality assessment

To assess risk of bias, the Systematic Review Center for Laboratory Animal Experimentation (SYRCLE) tool for animal studies was used^11^. It contains the following assessment categories: selection, performance, detection, attrition, reporting, and other sources of bias. Overall, 10 questions were applied to the articles in this systematic review, the answers to which could be “YES” (indicating a low risk of bias), “NO” (indicating a high risk of bias), and “UNCERTAIN” (indicating an uncertain risk of bias). It is not recommended to calculate the sum score of each study using this tool.

## RESULTS

### Characteristics of the included studies

**Figure 2:**
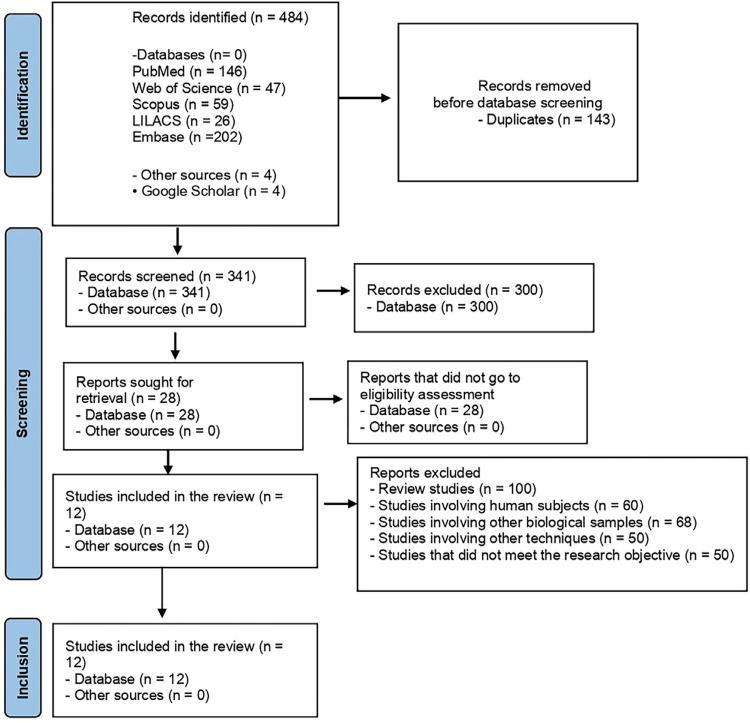
PRISMA 2020 flow diagram.

The search strategies in this review retrieved 484 studies in the searched electronic databases (Figure 2) following the PRISMA criteria^7^. After the initial reading of the titles and abstracts, this review chose 341 studies for full reading. The inclusion/exclusion criteria only rendered 12 studies as eligible for this systematic review.

Table 1 shows the main characteristics of the 12 studies in this systematic review^12-23^ The articles were published from 2014 to 2024 in five continents and aimed to detect *Plasmodium* in anopheles using molecular biology techniques.

**Table 1:** Main characteristics of the studies in this systematic review

Article	Place	Mosquito species	Number of pools	Number of mosquitoes	Collection period
Molina Gomez *et al*.^12^	Colombia	*An. Apicimacula, An. Darlingi, An. Nuneztovari, An. Triannulatus.*	Not informed	1305 mosquitoes	2015 and 2016
Tadesse *et al.* ^13^	Ethiopia	*An. arabiensis.*	Not informed	406 mosquitoes	2017
Cunninghamm*et al.* ^14^	United States, Thailand, Uganda, and Democratic Republic of Kongo	*An. dirus*	10 pools	92 mosquitoes	2020 and 2021
Tadesse *et al.* ^15^	Ethiopia, Netherlands, and United Kingdom	*An. arabiensis*	Not informed	399 mosquitoes	October to December 2016
Hsiang *et al.* ^16^	United States	*An. arabiensis*	Not informed	10890 mosquitoes	October to May 2012 to 2015
Mirahmadi *et al.* ^17^	Iran	*An. stephensi*	Not informed	360 mosquitoes	July to December 2018
Timinao *et al.* ^18^	Papua New Guinea	*An. farauti*	36 pools	360 mosquitoes	2021
Waisberg *et al.* ^19^	United States, Brazil, and Czech Republic	*An. gambiae*	3 pools	60 mosquitoes	2013
Nkemngo *et al*.^20^	Cameroon	*An. gambiae and An. funestus*	Not informed	176 mosquitoes	March to August 2021
Raddi *et al.* ^21^	United Kingdom and United States	*An. gambiae*	10 pools	100 mosquitoes	2019
O’Meara *et al.* ^22^	Kenya	*An. gambiae, An. funestus, An. demeilloni, An. pretoriensis*	Not informed	1494 mosquitoes	July 2017
Mirahmadi *et al.* ^23^	Iran	*An. Culicifacies, An. dthali, A. stephensi*	Not informed	400 mosquitoes	March 2018 to July 2019

Thus, database search strategies provided a reliable basis for a broad analysis of molecular biology results, enabling the detection of *Plasmodium* and of various molecular diagnostic pathways. Moreover, they used new mechanisms to aid in the control of parasitic diseases, particularly malaria, and in monitoring the spread of the disease and the propagation of the vector^24^.

The mosquitoes in Table 1 refer to the prevalence of specimens in geographic areas with an endemic importance for the incidence of human malaria. All studies aimed to collect mosquitoes in areas with recorded malaria transmission.

The studies aimed to capture female mosquitoes due to their hematophagy (the act of sucking blood in search of the necessary nutrients for reproduction viability), inoculating *Plasmodium* in humans and animals, causing diseases^25^.

Mosquitoes are taxonomically classified in the order Diptera, the suborder Nematocera, and the family Culicidae, comprising about 3,618 described species. Thus, eligible studies targeted the *Anopheles* genus because these vectors can transmit malaria to humans and vertebrate animals and for their considerable vectorial capacity^3,26^.

All eligible studies showed the effectiveness of molecular biology techniques to assess the vector competence of *Anopheles* mosquitoes in malaria transmission. Furthermore, the sampled regions showed considerable species variability, including species restricted to specific countries and continental areas, which show greater affinity with the etiological agent of malaria.

### Malaria around the world

The incidence of malaria is more concentrated in the tropical and subtropical regions of the globe since these regions have ecological and climatic characteristics that are conducive to mosquito breeding grounds and the circulation of *Anopheles*.

Statistical and epidemiological data from the World Health Organization show that, despite a global decline in malaria cases, this parasitic disease remains one of the most significant public health burdens. Moreover, it is important to consider that underreporting and deaths not attributed to malaria often occur in many countries, hindering epidemiological monitoring and surveillance systems^1^.

The parasitic species that transmit malaria vary according to the characteristics of each region, as in Table 2.

**Table 2:** Distribution of prevalent *Plasmodium* species across global regions

Region	Variant	Pathogen
African	Tropical Malaria	*Plasmodium falciparum* and *ovale*
South and Central America	*Terçã* Malaria	*Plasmodium vivax*
Southeast Asia	*Quaternã* Malaria	*Plasmodium knowlesi*
Worldwide	*Quaternã* Malaria	*Malariae*

Source: World Health Organization^1^.

These variants by categorization occur due to the transmission routes by pathogen (according to global epidemiological data). However, this system only serves as auxiliary data since they consist of general information about the parasitic species in each region, and may therefore undergo variations due to climatic, population, and phytophysiognomic factors^1^.

The most recent worldwide data on malaria shows a stable and decreasing incidence from 2000 to 2023. The number of infected people suggests that it remained relatively constant during this period, varying from 200 to 250 million cases per year. These numbers show small fluctuations, with a downward trend from 2004 to 2010 and an increase again in more recent years, particularly in 2016.

Death rates showed a sharp drop from 2000 to 2010, going from 800,000 to less than 600,000. However, from 2011 to 2023, deaths stabilized at 400,000 to 500,000 (with small fluctuations).

### Anopheles diversity and vectorial capacity

According to Molina Gomez *et al.* ^12^, a study in Colombia showed the effectiveness of reactive case detection (RACD) to find secondary malaria infections, highlighting the role of *An. nuneztovari* and *An. darlingi* mosquitoes in peri-urban areas. The used molecular biology techniques also included quantitative real-time PCR (qPCR) and ELISA, including the analysis of 1,305 found mosquitoes from 2015 to 2016 (Tables 1 and 2)^12^.

Tadesse *et al*.^13^ found *An. Stephensi*, number of mosquitoes (10,890) from 2012 and 2015; these mosquitoes (found in Ethiopia, the Netherlands, and the United Kingdom) showed high relevance for transmission and reinforced the importance of early diagnosis.

Studies in Iran found *Plasmodium vivax* and *Plasmodium falciparum* in *An. stephensi* mosquitoes using nested PCR. Such research pointed to the role of these mosquitoes in transmission in endemic regions from 2018 to 2019 (Tables 1 and 3)^17^.

Molina Gomez *et al.* ^12^ conducted a study in Colombia that highlighted the effectiveness of reactive case detection (RACD) in finding secondary malaria infections. The research highlighted the role of *An. nuneztovari* and *An. darlingi* mosquitoes in peri-urban areas, analyzing 1,305 mosquitoes collected from 2015 to 2016 using molecular biology techniques such as qPCR and ELISA (Tables 1 and 3)^12^.

Tadesse *et al*.^13^ reported that asymptomatic *Plasmodium vivax* and *Plasmodium falciparum* infections accounted for most mosquito-specific transmissions in Ethiopia, the Netherlands, and the United Kingdom. This study emphasized the importance of early diagnosis and treatment to reduce malaria reservoirs using methods such as PCR and membrane feeding assays^13^.

On the other hand, Mirahmadi *et al*.^17^ found *Plasmodium vivax* and *Plasmodium falciparum* in the *An. Stephensi* population in Iran. Using nested PCR, the authors highlighted the impact of these mosquitoes on transmission in endemic regions from 2018 to 2019.

### Molecular biology used in the detection of Plasmodium

The study by Tadesse *et al*.^15^ highlighted the importance of early diagnosis and treatment to reduce malaria reservoirs, using methods such as PCR and membrane feeding assays (Table 1 and 3). The main sequencers in the studies referred to NovaSeq, Miseq. and HiSeq (Illumina). Only one study used Ion Torrent S5 XL (Thermo Fisher Scientific, Waltham, MA, USA)^27^.

Raddi *et al*.^21^ used single-cell RNA sequencing to find novel cell subtypes, termed “megacytes,” in *A. gambiae* and *A. aegypti* mosquitoes. These cells play a fundamental role in the immune response against *Plasmodium*, expanding our understanding of the defense mechanisms of the vectors. The authors found that using isothermal DNA amplification (LAMP) to detect malaria more efficiently found hotspots in malaria-eliminated areas of the United States^16^.

Molina Gomez *et al.* ^12^, using qPCR, STR genotyping, and ELISA, showed the importance of *An. nuneztovari* and *An. darlingi* vectors in malaria transmission in peri-urban areas of Colombia. The authors applied qPCR, RT-PCR, and membrane feeding assays to study asymptomatic *Plasmodium vivax* and *Plasmodium falciparum* infections in *An. Arabiensis* ^13^.

Cunningham *et al*.^14^ have shown that CRISPR-SHERLOCK-based molecular diagnostics to differentiate *Plasmodium* species in *An. dirus* mosquitoes found in countries such as Uganda and Thailand were of great importance. Furthermore, the technique showed high sensitivity and specificity, suggesting its applicability in field diagnostic. Thus, the authors introduced an innovative molecular diagnostic based on the CRISPR-SHERLOCK technique to detect and differentiate *Plasmodium* species. This method showed high sensitivity and specificity in mosquitoes that were collected in countries such as Uganda and Thailand^14^.

Timinao *et al*.^18^ applied qPCR to detect of oocysts and sporozoites in *An. farauti*, showing that the heating method was more sensitive in identifying *P. vivax* in Papua New Guinea. They analyzed 360 mosquitoes in 2021.

Waisberg *et al*.^19^ used microarrays and Nanostring technology to evaluate gene expression in *An. gambiae* infected with *Plasmodium falciparum*. The study found anti-hemostatic proteins that favor the survival of the parasite, with mosquito samples from several continents. Investigating *An. gambiae* and *An. funestus* in Cameroon, the authors sequenced the dhfr and dhps genes. The research showed a high prevalence of asymptomatic *Plasmodium falciparum* infections and resistance to sulfadoxine-pyrimethamine in 176 mosquitoes^20^.

PCR has been crucial in enhancing vector analysis, particularly for identifying *Plasmodium* in *Anopheles* mosquitoes. Traditional methods, such as nested-PCR targeting the 18S rRNA gene, offer high sensitivity but require multiple steps, including post-PCR analysis via agarose gel electrophoresis^28^. In contrast, qPCR enables genetic material quantification with a lower risk of contamination, although its sensitivity depends on factors such as primer design and MgCl_2_ concentrations^29^.

In recent years, advanced alternatives have emerged to overcome the limitations of sensitivity and quantification. Droplet digital PCR (ddPCR) has detected *Plasmodium cynomolgi* DNA in *Anopheles* samples from Thailand for which nested-PCR failed, highlighting its capability to find low-density infections and previously unrecognized potential vectors^30^. Its high precision and resistance to inhibitors make it particularly suitable for complex epidemiological studies.

Comparative studies have shown the advantages of ddPCR over qPCR in detecting low parasitemia. A duplex assay targeting *P. knowlesi* and *P. vivax* showed a greater sensitivity of ddPCR, reinforcing its potential for investigating subclinical infections and confirming large-scale quantitative results^31^. These advancements are especially relevant in regions with mixed-species transmission in which multiple *Plasmodium* species may infect mosquito vectors.

Despite the advantages of ddPCR and qPCR, conventional PCR remains relevant due to its accessibility and cost-effectiveness in resource-limited control programs. Moreover, TaqMan probe-based multiplex assays have been developed to simultaneously detect multiple *Plasmodium* species in *Anopheles*, maintaining sensitivity while enabling high-throughput processing in preserved samples^28^.

Thus, the PCR-based detection of *Plasmodium* in anopheline mosquitoes has significantly evolved, offering accurate quantification, high sensitivity, and the ability to identify vectors harboring subclinical infections. Meanwhile, qPCR and multiplex PCR continue to serve as valuable tools for rapid screening and large-scale applications. Method selection should follow the intended purpose—whether molecular surveillance, etiological research, or the monitoring of control strategies—balancing accuracy, cost, and the available infrastructure^29,30^.

### Quality of evidence

Table 4 shows information regarding the quality of evidence/risk of bias based on the SYRCLE tool for animal studies^11^ and adapted according to the guidelines of this tool, which recommends that researchers and collaborators who are going to assess the risk of bias of the included studies discuss and adapt it to the specific needs of their review^11^.

**Table 4:** Study quality assessment according to the SYRCLE scale

Article	Selection bias			Performance Bias		Detection Bias		Friction Bias	Reporting Bias	Other sources of bias
	1	2	3	4	5	6	7	8	9	10
Molina Gomez^13^	N	Y	N	N	N	Y	Y	?	Y	Y
Tadesse *et al.* ^13^	N	Y	N	N	N	Y	Y	?	Y	Y
Cunningham *et al.* ^14^	Y	Y	N	N	N	Y	Y	?	Y	Y
Tadesse *et al*.^15^	N	Y	N	N	N	Y	Y	?	Y	Y
Hsiang *et al.* ^16^	N	Y	N	N	N	Y	Y	?	Y	Y
Mirahmadi *et al.* ^17^	N	Y	N	N	N	Y	Y	?	Y	Y
Timinao *et al.* ^18^	Y	Y	N	N	N	Y	Y	?	Y	Y
Waisberg *et al.* ^19^	Y	Y	N	N	N	Y	Y	?	Y	Y
Nkemngo *et al.* ^20^	N	Y	N	N	N	Y	Y	?	Y	Y
Raddi *et al.* ^21^	Y	Y	N	N	N	Y	Y	?	Y	Y
O’Meara *et al.* ^22^	N	Y	N	N	N	Y	Y	?	Y	Y
Mirahmadi *et al.* ^23^	N	Y	N	N	N	Y	Y	?	Y	Y

Y = Yes (indicates low risk of bias); N = No (indicates high risk of bias); ? = Uncertain (indicates unclear risk of bias).

This systematic review only included primary, experimental, cross-sectional, and prospective studies. Although this systematic review had limitations in terms of adequate methodological quality assessment, it classified all studies as having low risk of bias.

The selection bias in columns 1 and 2 in Table 4 refers to allocation sequences: all studies identified the mosquitoes by species but only three included information on allocation into groups (pools) (Table 1). Regarding basic characteristics, all articles standardized their groups by the number of specimens, storing them in refrigeration to preserve their viral DNA or RNA and then processing them for molecular biology analysis.

Item 3 showed a high risk of bias due to such evidence in its group allocation as it is essential to identify the mosquito species to understand the diversity and ecology of the viruses after genomic sequencing.

Regarding performance bias, columns 4 and 5 referred to random housing and blinding, respectively. All articles distributed their groups by species, refrigerating the samples without the need for random housing. Researchers performed no blinding as it is of utmost importance to have knowledge about the mosquito species. Therefore, they showed a high risk of bias.

When reporting on detection bias, column 6 addresses the random assessment of the outcome, carried out randomly according to the detection of *Plasmodium* in the samples. In column 7, blinding refers to the application of a molecular biology technique to detect the parasitic species. Both items showed a low risk of bias.

Regarding the attrition bias, item 8 refers to incomplete outcome results. No article mentioned excluding mosquito groups in outcome assessment. Thus, we classified this risk of bias as uncertain. Regarding reporting bias in item 9 (selective outcome reporting), all studies detected the presence of *Plasmodium*. Regarding other sources of bias (item 10), no article showed other sources of bias, evincing a low risk of bias.

## DISCUSSION

Malaria remains one of the major infectious diseases affecting tropical and subtropical regions. In recent years, research has focused on understanding transmission dynamics in several geographic contexts, especially on the interface between rural and urban areas. In Colombia, the study by Molina Gomez^12^ analyzed transmission in Quibdo, showing that RACD has greater effectiveness in peri-urban regions due to their higher concentration of asymptomatic cases and foci of *Plasmodium vivax* transmission. The research highlighted the importance of combining epidemiological and genetic approaches to find hidden patterns of transmission.

The interface between urban and rural areas plays a crucial role in the spread of malaria. In Colombia, significant socioeconomic differences between urban and peri-urban areas influence the distribution of the disease^12^. While urban areas have a more developed infrastructure, peri-urban regions have poor housing and proximity to mosquito breeding sites, facilitating the transmission of *Plasmodium vivax* and creating additional challenges for disease control.

A relevant point in Molina Gomez^12^ refers to the relevance of parasite genotyping to better understand transmission dynamics. This enabled the identification of genetically distinct primary and secondary cases, showing independent techniques in peri-urban areas. This approach complements traditional detection methods and enhances the efficiency of control programs.

In Ethiopia, Tadesse *et al*.^13^ investigated the contribution of symptomatic and asymptomatic infections to the infectious reservoir in a context of low endemicity. The results showed that most mosquito-borne evidence shows asymptomatic infections, even if submicroscopic. This highlights the need for the early detection and treatment of these infections to accelerate malaria elimination efforts.

Asymptomatic transmission represents a major challenge for malaria control. According to Tadesse *et al*.^13^, infections caused by *Plasmodium vivax* show greater infectivity for mosquitoes than those caused by *Plasmodium falciparum*. However, due to the high prevalence of asymptomatic infections, individuals perpetuate transmission, even in areas of low endemicity.

Furthermore, in low-density and asymptomatic areas, testing in blood banks is necessary. Hergott *et al*.^32^ have shown that the molecular diagnostic methods used to detect pre-existing and intervening *Plasmodium* in dried blood spot samples enable greater monitoring of more complex infections and provide support for future clinical trials and epidemiological studies on malaria.

Another critical aspect refers to the relation between parasite density and infectivity. In Ethiopia, symptomatic infections had higher parasite and gametocyte densities, significantly increasing the likelihood of transmission. These data reinforce the importance of integrated approaches that combine clinical and community surveillance to reduce transmission^13^.

Research findings in Colombia and Ethiopia highlight the need to adapt strategies to geographic and epidemiological contexts. In Colombia, actions should prioritize reducing mosquito breeding sites in peri-urban areas and improving its basic infrastructure. In Ethiopia, improving detection of asymptomatic infections and involving targeted interventions targeting infectious reservoirs are essential.

Diagnostic technologies play a vital role in identifying hidden infections. Methods such as molecular genotyping and quantitative PCR offer greater accuracy in detecting submicroscopic infections, improving malaria surveillance and control^12,13^.

In recent years, the surveillance and control of malaria have made important advances, especially in areas of low transmission. Hsiang *et al*.^16^ have evaluated the effectiveness of reactive case detection in Eswatini using LAMP. This approach can finding infections with thrice the efficacy of rapid and casual tests, highlighting the relevance of molecular methods in regions of low endemicity^16^. Strategies based on local data, such as the use of networks and household entomological surveillance, play a key role in reducing the malaria burden in vulnerable communities^22^.

Molecular methods also excel in finding *Plasmodium* species in malaria vectors. Nested PCR constitutes a highly sensitive tool to detect *P. vivax* and *P. falciparum* infections in *Anopheles* mosquitoes in Iran, reinforcing its value in endemic regions^17^. Advances in transcriptomics may open paths for manipulating vector immune responses. For example, the identification of markers such as LL3 in megacytes offers perspectives for new control strategies^21^.

Another crucial aspect in the study of malaria refers to understanding the mechanisms that facilitate the transmission of the parasite. Waisberg *et al*.^19^ found that *Plasmodium falciparum* infections induce the expression of salivary proteins in *Anopheles* mosquitoes such as *Agaphelina*. This protein initiates the action of neutrophils and prevents the formation of thrombi without compromising hemostasis. These findings suggest a complex interaction between vector, parasite, and human host, opening new possibilities for therapeutic interventions^19^. The co-endemicity of *Plasmodium* species challenges diagnostic differentiation. Methods such as qPCR, which use specific probes, may overcome these difficulties, as per Timinao *et al*.^18^

Drug resistance remains a critical challenge. Nkemngo *et al*.^20^ documented the increase in sulfadoxine-pyrimethamine-resistant alleles in human and mosquito populations in Cameroon. That study highlighted the high prevalence of resistant haplotypes, such as a quadruple mutant, evincing the urgent need for continued monitoring and adjustments in treatment policies^20^.

This resistance also impacts the effectiveness of preventive strategies, such as intermittent preventive treatment during pregnancy. Recent reports warn that resistant variants may compromise the success of these interventions in areas of high transmission^28^.

Furthermore, logistical challenges in implementing control measures such as RACD remain significant. Hsiang *et al*.^16^ emphasized that factors such as population coverage and response time are crucial to the success of RACD, highlighting the importance of resources and adequate training^16^.

The literature has explored innovative strategies, such as molecular tools for genomic surveillance, to address resistance. These tools are crucial for tracking the spread of resistant alleles and adjusting interventions in real time, in line with World Health Organization guidelines^28^. Integrating high-resolution technologies such as transcriptomics and qPCR with traditional approaches could revolutionize malaria control, making it more efficient and adapted to local needs^22,32^.

The integration of molecular and epidemiological data may also improve discipline planning. For example, Mirahmadi *et al*.^17^ suggest that nested PCR identifying infections and helps to map the distribution of vectors and parasites, providing strategic information for local control.

Another relevant point refers to the impact of resistance on vectors. According to Nkemngo *et al*.^20^, resistance can influence the life cycle of the mosquito parasite, with infection rates varying significantly between different *Anopheles* species. Timinao *et al*.^18^ highlighted that the use of qPCR, associated with the heating method for DNA extraction, offers superior sensitivity in the detection of *P. vivax* oocysts and sporozoites. This reduces costs and processing time, being especially useful in transmission studies.

Despite its high performance, qPCR faces challenges such as the need for specialized equipment and relatively expensive reagents. More accessible methods, such as the heating protocol for DNA extraction, are important steps toward widespread application in resource-limited contexts^18^.

Analyzing the relations between infections and death rates shows evidence of a decline in the number of deaths, suggesting advances in treatment and prevention, especially in the use of mosquito nets and effective medications. Although the number of deaths has decreased, infection rates remain high, highlighting that malaria remains a global public health problem.

To further reduce these numbers of infected people and deaths, continuous action measures are necessary to maintain and intensify efforts to combat the vector agent and consequently reduce malaria.

### Molecular techniques and their application in epidemiology and vector control strategies

Modern molecular techniques, especially those based on PCR and sequencing, have revolutionized the epidemiology of infectious diseases by enabling rapid, sensitive, and quantitative diagnostics. QPCR can precisely detect and quantify pathogens in clinical or environmental samples, reducing the risk of post-amplification contamination and enabling large-scale analysis of viral or bacterial loads. These features have made qPCR the gold standard in viral surveillance, as in the COVID-19 pandemic, during which it played a key role in epidemiological tracking and rapid public health response^33^.

In recent decades, next-generation sequencing (NGS) has emerged as a key tool for pathogen characterization and epidemiological surveillance. Whole genome sequencing (WGS) can trace lineages, understand antimicrobial resistance, and map transmission chains in hospital and community settings with extremely high resolution. Studies have shown that, in MRSA and *E. coli* outbreaks, WGS has employed to identify sources and transmission spillovers that classical typing techniques failed to detect^34^.

PCR-based amplicon NGS methods combine the specificity of primers with the high throughput power of sequencing to detect emerging variants, monitor viral loads, and track viral evolution in real time. The surveillance of SARS-CoV-2 widely used this approach, identifying variants even in environmental samples, such as wastewater, and informing public health decisions^35^. Moreover, studies on HIV and HCV have shown that near real-time NGS can find transmission clusters and support rapid interventions^36^.

Alternatives such as ddPCR and isothermal PCR (e.g., LAMP, RPA) have shown advances in low-resource settings. DdPCR offers absolute quantification with high sensitivity for rare mutations and low pathogen loads^34^. Although isothermal techniques may fail to achieve the same amplification levels as conventional PCR, they are robust and field-adapted—particularly in the surveillance of diseases in tropical regions.

The combination of methods—ranging from qPCR and ddPCR to WGS—has strengthened molecular epidemiology within the “One Health” paradigm, integrating human, animal, and environmental surveillance. This integration is critical in tropical areas with emerging zoonoses, facilitating early outbreak detection and cross-species transmission monitoring^34^.

Furthermore, molecular techniques have significantly impacted vector control by allowing accurate species identification and insecticide resistance monitoring. In Angola, Alves *et al*.^37^ used PCR to find *Anopheles* species and detect resistance mutations (kdr), which are essential for defining zones in which chemical control is ineffective. These methods provide greater sensitivity than morphological separation, improving targeted intervention strategies.

In genetic engineering, tools such as CRISPR/Cas9 with gene drive are emerging as revolutionary strategies for mosquito population suppression. Apte *et al*.^38^ have described a pgSIT system that induces male sterility and female lethality in *Anopheles gambiae*, resulting in nearly 100% sterility in simulated field trials. This approach offers species-specific control and significantly reduces vectorial capacity.

Modeling studies have also supported the viability of these molecular tools. North *et al*.^39^ have carried out spatial simulations of a gene drive targeting the doublesex gene, showing that resistance mutations emerge slowly and that population suppression can persist over thousands of square kilometers. This modeling evidence suggests that gene drives can be effective at large scales under various ecological conditions.

Despite the emergence of advanced molecular technologies, classical PCR methods remain fundamental to vector surveillance. Reichl *et al*.^40^ highlight that multiplex PCR can detect resistance mutations and *Plasmodium* infections while simultaneously finding vector species. This versatility enables public health programs to integrate vector and pathogen monitoring in a cost-effective and reliable manner.

Finally, the advancement of gene-editing techniques continues to expand horizons by offering alternatives such as gene drive, pgSIT, and CRISPR/Cas9, with greater sustainability appeal. Apte *et al*.^38^ and Reichl *et al*.^40^ emphasized recent improvements in delivery systems such as ReMOT control and off-target evaluation, making these techniques increasingly safe and specific.

## CONCLUSION

Finally, managing malaria requires interdisciplinary efforts. From basic research to health program implementation, it is essential to integrate initiatives to overcome resistance challenges and expand access to diagnostics and therapeutic treatments.

The studies in this review show that technological advances and personalized approaches can transform malaria control. However, it is essential to integrate these innovations with sustainable, local, evidence-based strategies to maximize their impact.

**Table 3:** Main findings of the eligible studies in this systematic review.

Article	Molecular Biology Technique.	Main results of *Plasmodium* detected with molecular biology.
Molina Gomez *et al*.^12^	qPCR, STR Genotyping, ELISA	The study showed the effectiveness of reactive case detection (RACD) to find secondary malaria cases and highlighted the role of *An. nuneztovari* and *An. darlingi* vectors in peri-urban areas.
Tadesse *et al*.^13^	qPCR, RT-PCR, Membrane Feeding Assay	Asymptomatic infections of *P. vivax* and *P. falciparum* caused the most transmissions to mosquitoes, suggesting the importance of early treatment to eliminate reservoirs.
Cunninghamm*et al*.^14^	CRISPR-based diagnostics (SHERLOCK)	Development of diagnostics for *Plasmodium* detection and species differentiation with high sensitivity and specificity that is comparable to PCR and deep sequencing
Tadesse *et al*.^15^	PCR and Membrane Feeding Assays	Asymptomatic infections are highly prevalent and significant for transmission, suggesting the need for identification and treatment to accelerate malaria elimination.
Hsiang *et al*.^16^	Isothermal DNA amplification (LAMP)	Reactive malaria case detection with LAMP improves hotspot efficiency, recommending LAMP over RDT for infection detection in malaria elimination settings
Mirahmadi *et al.* ^17^	Nested PCR	Detection of *P. vivax* and *P. falciparum* in *An. stephensi* populations in endemic regions.
Timinao *et al.* ^18^	qPCR for detection of oocysts and sporozoites	The chosen heating method increases the sensitivity in detecting *P. vivax* in infected mosquitoes.
Waisberg *et al*.^19^	Microarray and RNA quantification using Nanostring	*Plasmodium falciparum* infection in *Anopheles gambiae* induces the expression of anti-hemostatic proteins, such as *Agaphelin*, which inhibit neutrophil function without affecting hemostasis, suggesting an immune evasion mechanism for the parasite.
Nkemngo *et al.* ^20^	Sequencing of dhfr and dhps genes from *P. falciparum* and *P. malariae*	High frequencies of sulfadoxine-pyrimethamine resistant mutant alleles in mosquitoes and humans and the high prevalence of asymptomatic *P. malariae* and *P. falciparum* infection in Cameroon. It highlights the need for continued surveillance of drug resistance.
Raddi *et al*.^21^	Single-cell RNA sequencing (scRNA-seq)	The functional diversity of hemocytes in *A. gambiae* and *A. aegypti* showed granulocyte subtypes with several gene expression profiles. It introduced the term “megacytes” as a new cell type. These hemocytes played a key role in the immune response to *Plasmodium* infections.
O’Meara *et al*.^22^	PCR for detection of parasites and human blood in mosquitoes	Malaria risk has been directly associated with exposure to sporozoite-infected mosquitoes. Exposure to vectors has been shown to increase malaria risk, but the use of insecticide-treated nets significantly reduces the sensitivity of malaria risk to vector density.
Mirahmadi *et al*.^23^	LAMP and nested PCR (Multiplex Nested-PCR)	It found *Plasmodium vivax* in 1.5% of the collected mosquitoes, showing the effectiveness of LAMP as a rapid and practical method for field diagnosis.

## Data Availability

The complete anonymized dataset supporting the findings of this study is included within in the article itself.
